# Editorial: Recent advances in papillary thyroid carcinoma: diagnosis and predictive factors

**DOI:** 10.3389/fendo.2023.1283397

**Published:** 2023-09-15

**Authors:** Erivelto Martinho Volpi, Margarita Carmen Ramirez-Ortega, Jose Federico Carrillo

**Affiliations:** ^1^ Head and Neck Department, Centro de Referencia no Ensino do Diagnóstico por Imagem (CETRUS), São Paulo, Brazil; ^2^ Departamento de Farmacología, Instituto Nacional de Cardiologia Ignacio Chavez, Mexico City, Mexico; ^3^ Head and Neck Department, National Institute of Cancerology (INCAN), Mexico City, Mexico

**Keywords:** differentiated thyroid carcinoma, TERT (telomerase reverse transcriptase) in thyroid carcinoma, Radioiodine resistance in differentiated thyroid carcinoma, CEUS (Contrast enhanced ultrasound) in thyroid cancer, BRAFV600 mutation in thyroid carcinoma, FNAB (fine needle aspiration biopsy) in thyroid nodules

In this Research Topic, novel strategies are presented to discern genomic and molecular basis, early and accurate diagnosis of Papillary thyroid carcinoma(PTC), as well as predictive factors for recurrence, which help in the design of new treatment strategies. **Figure 1**.

**Figure 1:**
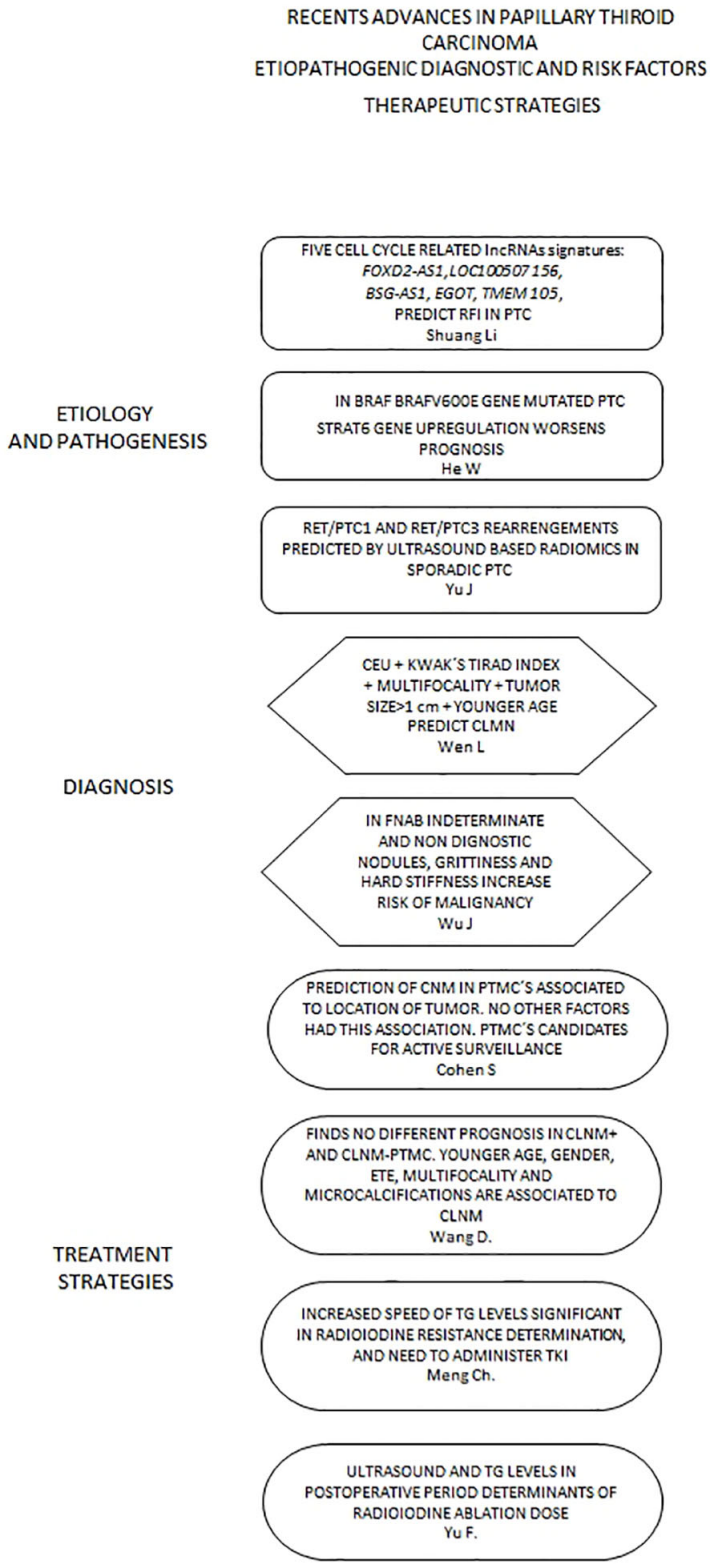
Presents a flow chart that displays relevant articles included in this topic according to different scopes regarding Papillary Thyroid Carcinoma. lncRNAs, long non-coding protein RNA; RFI, Recurrence Free Interval; PTC, Papillary Thyroid Carcinoma; CEU, Contrast-enhanced Ultrasound; CLNM, Central Lymph Node Metastasis; CNM, Cervical Node Metastases; FNAB, Fine Needle Aspiration Biopsy; PTMC, Papillary Thyroid Microcarcinoma; ETE, Extrathyroid Extension; TG, Thyroglobulin; TKI, Thyrosine Kinase Inhibitors.

Cell cycle-related lncRNAs (long non -coding protein RNA) signatures (CCLRSig) are investigated by Li et al., utilizing the TCGA. They identify five signatures (FOXD2-AS1, LOC100507156, BSG-AS1, EGOT, and TMEM105) which predict the recurrence-free interval (RFI) in papillary PTC through Kaplan-Meier analyses, Receiver Operating Curve (ROC), and a Cox multivariate model. Further, a nomogram with clinicopathological features included gave an area under the curve (AUC) in ROC analyses, of 0.681 at five years. The higher risk scores determined by the aforementioned signature correlated with higher immune cell infiltration patterns which suggests a regulatory role from their findings to the immune processes of the malignancy.

Another paper by He et al., defines, with data extracted from the TCGA - in BRAFV-600 gene mutated thyroid carcinomas -STRA6 gene upregulation which defines the nature of the immune system activation and interaction of IL2-STAT5 pathway. Decreased activation of CD8+ T cells in the tissue harboring these thyroid carcinomas defines a higher potential for invasion and metastasis development, involving an increase in the epithelial-mesenchymal transition process, all of which worsens the prognosis of patients with upregulated STRA6. These facts open a new venue for the development of more precise therapeutic strategies.

Regarding genetics encoding papillary thyroid carcinoma (PTC), Li et al. analyzed patients with stages I and IV, comparing different stages of PTC using The Cancer Genome Atlas(TCGA) with edgeR technology and describe TIMP1, LOX, CD276, IFNA1, TLR2, and POSTN as key genes involved in the development of the malignancy. As well, the protein-protein interaction (PPI) of these genes was evaluated and finally, 47 miRNA and 44 transcription factors were described bound to the previously described genes. CD276, POSTN, and IFNA1 may be considered as new potential biomarkers associated with the prognosis of thyroid cancer. In addition, TF–miRNA–target gene regulatory is involved in the development of PTC. These findings explain different clinical courses in patients with similar histology features and PTC.

Yu et al., through ultrasound images (Radiomics) construct a nomogram to predict RET rearrangements in sporadic papillary thyroid carcinoma. Although non-selective determination of RET/PTC1 and RET/PTC3 is performed, future efforts and studies are warranted to describe mutations associated with high-risk characteristics (RET/PTC3) without the need for a gene test.

An article by Fang et al. describes a combination of contrast-enhanced ultrasound (CEU) characteristics in thyroid nodules, to increase the diagnostic accuracy of this method in partially cystic lesions. Briefly, the peak intensity index (P1<1), centrifugal perfusion, and heterogeneous enhancement were predictors of malignancy, with better results compared to Kwak´s Ti-Rads index; however, the cases number is small and the study was performed by only one individual, which warrants prospective and larger studies including different operators.

In the same order of ideas, Li et al. describe the usefulness of CEU combined with primary tumor ultrasound characteristics to predict the presence of clinical (cN1a) and pathologic central lymph node metastasis (pN1a). They find tumor size >1cm, multifocality, and younger age combined with hyperenhancement in areas close to the primary tumor as indicative of cN1a metastases, which if validated in larger studies could lead to new paradigms regarding indications for central lymph node dissection. Regarding pN1a lesions, these were associated with young age, large size, and multifocality.

Prediction of BRAF mutations according to elastographic and gray-scale features of thyroid nodules ultrasound (although not including benign lesions) is reported by Wang et al. Although nowadays BRAF^v600^-mutation associated to high-risk prognosis in differentiated thyroid cancer is debated (1), the association of the ultrasonographic features (Radiomics) to the presence of this mutation is suggested and probably, in future studies, the association of these characteristics to other mutations like TERT (telomerase reverse transcriptase) promoter mutations chr-5, 1,295,228 C>T (C228T) and 1,295,250 C>T (C250T)) (2), will help to design more accurate surgical approaches and adjuvant therapies.

Although containing a small number of cases, the report from Xue et al. finds a color doppler ultrasound characterized by a spoked-wheel figure in thyroid nodules to be highly specific to detect papillary carcinoma, which has a 100% accuracy compared to grey-scale ultrasound (16%). This paper represents another interesting contribution to detect non-invasively the presence of thyroid cancer, and likely high-risk prognostic factors, although the prevalence of spoke-wheel blood flow is low in thyroid nodules (0.1%).

The use of Computerized Tomography (CT) Radiomics combined with clinical features of thyroid nodules to predict extrathyroidal extension (ETE) is described by Yu et al. They describe age, gender, tumor size, and a Rad-score (CT radiomics) as associated with ETE with an AUC (area under the curve) greater than 0.75 in the Receiver Operating Curve (ROC) analyses, which demonstrates a high efficiency for this nomogram. Larger studies on this subject are warranted and designed to obtain definitive external validation and improvement of this nomogram.

Regarding the prognosis of papillary microcarcinoma in T1N0M0 Stage, Qian et al. report, with the propensity score-matched (PSM) analyses, similar prognoses for patients treated with and without surgery in a series obtained from the SEER database, which adds support to the active surveillance strategy in these lesions, although the retrospective nature of the study, the lack of some clinical and pathological data expected from cases analyzed from the SEER and the small number of patients which did not have surgery, although elective candidates, warrant the need to perform larger and prospective studies.

Another clinical study by Zhao and Gong explores the SEER database and a PSM analyses the long-term effect of radioactive iodine (131I) in patients with low and intermediate risk. Although a retrospective study extracted from the SEER database, a beneficial effect was demonstrated on the whole with the administration of I131, specifically in patients >55 years of age, with multifocality and extrathyroid extension. In microcarcinomas, N0, unifocal and no extrathyroid extension cases the effect of radioiodine is nil.

ine needle aspiration biopsy (FNAB) of thyroid nodules has high accuracy; however, in spite of the TIRADs score and Bethesda system development, the presence of indeterminate and non-diagnostic categories in significant and cellular punctures, confers higher relevance to clinical findings as has been reported in previous studies (3). The paper from Wu et al. brings over again the subject regarding nodule clinical characteristics during FNAB procedures, and they find grittiness and hard stiffness as major aids in making decisions regarding nodules with indeterminate diagnosis. These facts warrant future large prospective studies to include the combination of FNAB, clinical, and radiologic factors to increase the diagnostic accuracy of this test, especially in the aforementioned Bethesda category.

Zhang et al. describe in a cohort of patients aged ≥65 years a nomogram that included ten factors: age, gender, marital status, histologic type, grade, TNM stage, surgery status, and tumor size, and predicted overall survival (OS) in their cohorts either in training and in external validation. This nomogram allowed the development of software that accurately predicted OS in geriatric patients. Surgery added better OS in high-risk patients. Although other nomograms (4) have been developed the application proposed is of major value in this specific population.

Eng et al. reports-with the identification of single nucleotide variants- progression of BTG (Benign thyroid goiter) to papillary thyroid carcinoma through FANCD1 (BRCA2) and FANCD2 genes, and hypothesize that papillary thyroid cancer related to goiter (PCb) and non-related to goiter (PCa) have different pathways of development. PCb was induced by Jak-STAT and Notch signaling pathways and PCa by thyroid kinase pathways activation. They propose independent mechanisms for the development of papillary thyroid cancer in patients with BTG, stating that PCb is not an intermediate stage in the development of thyroid cancer.

A very interesting report by Wang et al. performs a multivariate analysis of clinical factors and ultrasound characteristics to predict cervical node metastasis (CNM). Age, gender, nodule size, multifocality, contact extent with thyroid capsule ≥25%, maximal elastography ≥48.4, and interrupted capsule at CEUS in the multivariate analyses allowed calculation of CNM in thyroid carcinoma, with a ROC (Receiver Operating Curve) analyses with more than 80% predictive capability. Further studies are warranted for external validation of these results.

A clinical and genetic markers nomogram was created and validated by Ma et al. where they predict the presence of central lymph node metastases (CLNM) in papillary carcinoma patients clinically and ultrasonographically negative for CNM. Gender, Hashimoto´s thyroiditis, extrathyroidal extension, presence of TERT promoter, and NRas mutations were found significantly associated with CLNM. Of note, this study identifies as well the non-significant association of BRAFV600 mutation to risk factors and overall prognosis in papillary thyroid carcinoma.

A paper by Cohen et al. describes the prognostic factors of Papillary thyroid microcarcinoma (PTMC) associated with CNM and consequently its candidacy for active surveillance. They only find the location of the tumor either superior or inferior pole lesion as definers of CNM. Size, irregular margins, closeness to the capsule of the trachea and thyroiditis were non-associated to locoregional metastases. As an aside, in their study they consider microcarcinomas as nodules up to 2.7 cm and 1 cm in ultrasound and pathology diagnosis, respectively, and propose a definition of microcarcinomas further for lesions up to 1.5-2 cm by ultrasound.

A study in the same line by Wang et al. constructs a nomogram for the prediction of CLNM in PTMC. These authors find -using ultrasound, clinical, and pathological factors -that younger age, gender, ETE, multifocality and microcalcifications are the most significant ones associated with the presence of CLNM. Of note, DFS (disease-free survival) did not show a significant difference when comparing CLNM+ and CLNM- patients, which gives further support to the active surveillance policy in PTMC cases.

CT (Computerized Tomography) has not been considered a major tool for diagnosing CNM. An innovative article by Zhu et al. uses different contrast enhancements determined by CT in HU(Hounsfield Units), regarding lymph nodes(ENHU), esternocleidomastoid muscle(EMHU), and plain lymph node(PNHU) scans. They find that values of ENHU, ENHU-PNHU, ENHU-EMHU, and ENHU/EMHU are significantly related to high accuracy regarding detection of CNM with receiver-operating curve (ROC) analyses performed with higher than 0.8 area under the curve. This is one of the few manuscripts yet to exist regarding the use of CT in early- stage thyroid carcinoma.

Radioactive iodine resistant (RAIR) cases with metastatic disease are analyzed according to thyroglobulin and TSH change speed in the manuscript by Meng et al. Although the concept has been raised in previous reports and is considered by some authors as an important criterion to determine radioactive iodine-resistant malignancies (5), they construct a nomogram based on Thyroglobulin stimulated (Ts) differences at ablation and first recurrence: Ts1-Ts2: ΔTgs, as well as Thyroid Stimulant Hormone levels at same periods: TSHs1-TSH2= ΔTSHs. A ratio ΔTgs/ΔTSHs considering these two differences ≤1.5 as well as older age predicts rapid progression of the disease for these patients, which makes closer monitoring imperative, along with considering early administration of TKI agents.

The study by Yu et al., —although not including the extrathyroid extension factor (ETE)—designs a nomogram with two variants including overall ultrasound characteristics (first variant) and seven precise ultrasonographic signs (aspect transverse ratio, cystic change, microcalcification, mass hyperecho, echogenicity, lymphatic hilum structure, and vascularity) which together with Tg and TgAb levels are associated to residual disease or CLNM. This analysis presents a worthwhile tool to determine the best ablation radioiodine dose.

## Author contributions

EV: Conceptualization, Formal Analysis, Investigation, Project administration, Supervision, Visualization, Writing – original draft, Writing – review & editing. MR-O: Conceptualization, Data curation, Formal Analysis, Investigation, Methodology, Visualization, Writing – original draft, Writing – review & editing. JC: Conceptualization, Data curation, Formal Analysis, Methodology, Project administration, Supervision, Validation, Visualization, Writing – original draft, Writing – review & editing.

## References

[1] Henke LE Pfeifer JD Ma C Perkins SM DeWees T El-Mofty S. BRAF mutation is not predictive of long-term outcome in papillary thyroid carcinoma. Cancer Med (2015) 4(6):791–9. doi: 10.1002/cam4.417 PMC4472201 25712893

[2] Liu R Xing M. TERT promoter mutations in thyroid cancer. Endocr Relat Cancer (2016) 23(3):R143–55. doi: 10.1530/ERC-15-0533 PMC4750651 26733501

[3] Carrillo JF Frias-Mendivil M Ochoa-Carrillo FJ Ibarra M. Accuracy of fine-needle aspiration biopsy of the thyroid combined with an evaluation of clinical and radiologic factors. Otolaryngol Head Neck Surg (2000) 122(6):917–21. doi: 10.1016/S0194-59980070025-8 10828810

[4] Wang J Zhanghuang C Jin L Zhang Z Tan X Mi T. Development and validation of a nomogram to predict cancer-specific survival in elderly patients with papillary thyroid carcinoma: a population-based study. BMC Geriatr (2022) 22(1):736. doi: 10.1186/s12877-022-03430-8 36076163 PMC9454205

[5] Mutsuddy P Jeon S Yoo SW Zhang Y Chowdhury MSA Kim J. Optimization of serum thyroglobulin measured at different time points for prognostic evaluation in differentiated thyroid carcinoma patients. Med (Baltimore) (2020) 99(14):e19652. doi: 10.1097/MD.0000000000019652 PMC7440056 32243397

